# A novel compound heterozygous variant in *ALPK3* induced hypertrophic cardiomyopathy: a case report

**DOI:** 10.3389/fcvm.2023.1212417

**Published:** 2023-06-15

**Authors:** Tiange Li, Yuxi Jin, Rui Liu, Yimin Hua, Kaiyu Zhou, Shuhua Luo, Yifei Li, Donghui Zhang

**Affiliations:** ^1^ Key Laboratory of Birth Defects and Related Diseases of Women and Children of MOE, Department of Pediatrics, West China Second University Hospital, Sichuan University, Chengdu, China; ^2^ Department of Cardiovascular Surgery, West China Hospital, Sichuan University, Chengdu, China; ^3^ Department of Nursing, West China Second University Hospital, Sichuan University, Chengdu, China; ^4^ State Key Laboratory of Biocatalysis and Enzyme Engineering, School of Life Science, Hubei University, Wuhan, China

**Keywords:** *ALPK3*, hypertrophic cardiomyopathy, novel variant, case report, whole-exome sequencing

## Abstract

**Background:**

Malignant hypertrophic cardiomyopathy (HCM) phenotypes have potential risks of severe heart failure, fatal arrhythmia, and sudden cardiac death. Therefore, it is critical to predict the clinical outcomes of these patients. It was reported recently that the alpha kinase 3 (*ALPK3*) gene was involved in the occurrence of HCM. Herein we reported a girl with HCM, while whole-exome sequencing found novel compound heterozygous variants in *ALPK3* gene, which identified a potential association.

**Case presentation:**

We reported a 14-year-girl who suffered from clinical manifestations of cardiac failure, with sudden cardiac arrest before admission. The heartbeat recovered after cardiopulmonary resuscitation, though she remained unconscious without spontaneous breath. The patient stayed comatose when she was admitted. Physical examination indicated enlargement of the heart boundary. Laboratory results revealed a significant increment of myocardial markers, while imaging demonstrated hypertrophy of the left heart and interventricular septum. Whole-exome sequencing (WES) identified a compound heterozygous variant in *ALPK3* gene consisting of c.3907_3922del and c.2200A>T, which was inherited from her parents. Both variants (p.G1303Lfs*28 and p.R734*) were disease-causing evaluated by MutationTaster (probability 1.000). The crystal structure of the complete amino acid sequence is predicted and evaluated by AlphaFold and SWISS-MODEL software (July, 2022), which revealed three domains. Moreover, both variants resulted in a wide protein-truncating variant and damaged protein function. Thus, a novel compound heterozygous variant in *ALPK3* associated with HCM was diagnosed.

**Conclusion:**

We described a young patient with *ALPK3*-associated HCM who experienced sudden cardiac arrest. Through WES, we identified a compound heterozygous variant in the *ALPK3* gene, c.3907_3922del and c.2200A>T, which were inherited from the patient's parents and resulted in a truncated protein, indirectly causing the symptoms of HCM. In addition, WES provided clues in evaluating potential risks of gene variants on fatal clinical outcomes, and the nonsense and frameshift variants of *ALPK3* were related to adverse clinical outcomes in HCM patients, which required implantable cardioverter defibrillator (ICD) timely.

## 1. Introduction

Cardiomyopathies constitute a diverse group of disorders with clinical and genetic heterogeneity that primarily affect the ventricular myocardium, resulting in impaired cardiac function and heightened morbidity and mortality. According to existing practice and guidelines, cardiomyopathies can be classified into five subtypes based on their clinical phenotypes, including morphologic and functional features: hypertrophic cardiomyopathy (HCM), dilated cardiomyopathy (DCM), restrictive cardiomyopathy (RCM), arrhythmogenic right ventricular cardiomyopathy (ARVC), and unclassified cardiomyopathy (1). Among children, HCM and DCM are the most frequently encountered cardiomyopathy phenotypes (2). HCM is distinguished by symmetric or asymmetric left ventricular hypertrophy, particularly in the interventricular septum, obstructing the left ventricular outflow tract (3). Additionally, HCM is an inherited disease, with a predicted prevalence of 1/500 in adulthood (4). Given the high prevalence of HCM, it is crucial to differentiate between benign and malignant phenotypes and predict the risk of cardiac failure and sudden cardiac death, as some patients may be asymptomatic. In contrast, others present with atrial fibrillation, dyspnea, chest pain, fatigue, or syncope (4–6). The challenge of HCM diagnosis lies not only in making a definitive diagnosis but also in predicting or assessing the heterogeneity of phenotypes and associated clinical outcomes that may require implantable cardioverter defibrillator (ICD) implantation or heart transplantation (7–9). Although the etiology of HCM is highly diverse, it can be summarized as genetic or environmental factors (10). With the rapid development of genetic sequencing, over 900 genes have been identified as involved in the pathogenesis of HCM, with dominant molecules in the sarcomere or sarcomere-associated proteins being implicated in an autosomal dominant manner (11). Recent research has utilized genetic or polygenetic scores to predict clinical risks of HCM on a molecular level, aiding in the clinical management of high-risk patients and guiding the administration of ICD implantation or interventional treatment. The decreasing cost of next-generation sequencing has significantly promoted the application of genetic assessments in cardiomyopathies. Furthermore, hundreds of newly identified HCM-related genes or variant sites have been recorded, providing evidence of the association between rare genetic variants and strategies for diagnosis or treatment.

The alpha kinase 3 (*ALPK3*) gene, located on chr15:85360587-85416710, is a member of the family of atypical protein kinases (12), recently has been implicated in some cases of HCM, highlighting its potential role in the disease (10, 13, 14). In previous studies, *ALPK3* has been identified as a potential factor associated with myocardial cell differentiation, and mice with functional deficiency of *ALPK3* exhibit significant ventricular hypertrophy (15, 16). The *ALPK3* protein consists of two immunoglobulin (Ig)-like domains and an alpha-type protein kinase domain. Although its specific function in the heart remains unclear, it is believed to play a critical role in cardiac development and transcriptional regulation (10, 17). In this report, we present a case of a 14-year-old female with HCM who initially presented with symptoms of heart failure and experienced multiple cardiac arrests. Whole-exome sequencing (WES) revealed novel compound heterozygous variants on the *ALPK3* gene, underscoring the importance of ICD placement in *ALPK3*-related HCM patients. Furthermore, we provide a comprehensive review of the existing literature and discuss the molecular function of the *ALPK3* protein.

## 2. Case presentation

### 2.1. History of illness and physical examination

The study was approved by the ethics committee of the West China Second Hospital of Sichuan University (approval no. 2014–034). In addition, we obtained written, informed consent from the patient's parents prior to performing WES and for the inclusion of the patient's clinical and imaging details in publications.

The proband was a 14-year-old female who presented with a two-year history of reduced tolerance to physical exertion and subsequently experienced severe dyspnea, respiratory distress, and fatigue following exertion. The patient suffered a sudden cardiac arrest 2 h before arrival at the hospital, during which carotid pulsation and respiratory movement were absent. CPR and defibrillation were promptly administered by first-aid personnel, resulting in the return of heartbeats after 15 min and restoration of sinus rhythm. Nevertheless, the patient remained unconscious and exhibited no spontaneous breathing. The patient was transferred to the emergency department while receiving laryngeal mask ventilation and subsequently underwent tracheal intubation, positive pressure ventilation, fluid infusion, sedation, and analgesia. The patient was later transferred to the cardiac intensive care unit one hour after the cardiac arrest. The patient's parents denied any history of illness, especially cardiovascular disorders, and any family history of cardiac arrest or cardiovascular disease. Notably, no family member had a history of hypertension or coronary artery disease.

Upon arrival at the cardiac intensive care unit, the patient's blood pressure was approximately 92/63 mmHg, and arterial oxygen saturation was maintained at 97% through mechanical ventilation. Physical examination revealed the patient to be comatose with significant cardiac enlargement, thoracolumbar scoliosis, and muscle weakness.

### 2.2. Laboratory and imaging evaluation

The results of the blood gas analysis indicated extremely severe respiratory alkalosis (pH = 7.52, PCO2 = 24 mmHg, PO2 = 155 mmHg), electrolyte disturbance (K^+^ = 4.0 mmol/L, Na^+^ = 136 mmol/L, Cl^−^ = 104 mmol/L, and Ca^2+^ = 0.99 mmol/L), and high lactate levels (4.8 mmol/L). Peripheral blood counts revealed an increased leukocyte count of 13.2 × 109/L. Blood biochemical tests demonstrated an elevated level of lactic dehydrogenase [494 U/L; normal range (NR) 109–245 U/L], while other renal and hepatic function parameters showed no apparent abnormalities. Thyroid function test results showed a decreased free triiodothyronine level at 3.2 pmol/L (NR > 4.3 pmol/L). Myocardial markers revealed significantly increased levels of troponin I (0.791 µg/L; NR < 0.2 µg/L), creatine kinase MB isoenzyme (11.75 µg/L; NR < 5 µg/L), and b-type natriuretic peptide (>5,000.00 pg/ml; NR < 100 pg/ml).

**Figure 1:**
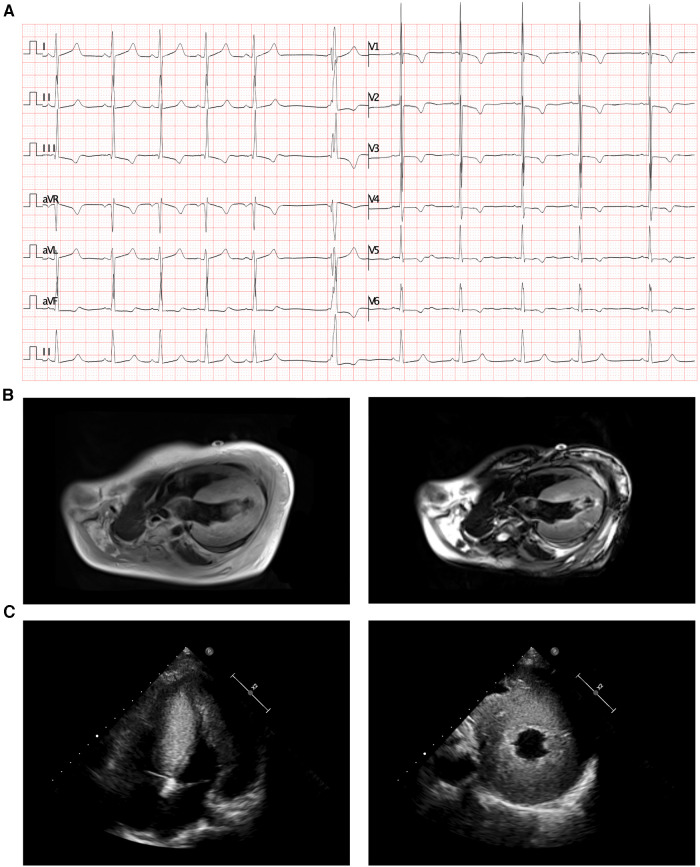
Radiology manifestation in the current proband. (**A**) Electrocardiographic examination demonstrated right axis deviation, enlargement of bi-ventricles and ventricular escape (arrow). (**B**) Cardiac magnetic resonance (CMR) demonstrated diffuse hypertrophy of ventricular myocardium, T2-weighting imaging revealed abnormal signal intensity of left ventricular subendocardial myocardium. (**C**) Transthoracic echocardiography (TTE) demonstrated significant hypertrophy of left ventricle and the interventricular septum.

The electrocardiogram (ECG) revealed right axis deviation, biventricular hypertrophy, and ventricular escape beats (Figure 1A). Transthoracic echocardiography revealed significant hypertrophy of the left ventricular wall, particularly the interventricular septum (IVS) and left posterior ventricular wall (LVPW) (Figure 1C). In addition, cardiac magnetic resonance imaging (CMR) demonstrated diffuse hypertrophy of the ventricular myocardium. The thickness of each part during the diastolic period was as follows: LVPW, 28.3 mm; right ventricular anterior wall (RVAW), 8.3 mm; and IVS, 32.2 mm (Figure 1B, left panel). Additionally, the left ventricular ejection fraction (LVEF) measured by CMR was decreased (40.2%). Furthermore, the T2-weighted image revealed an abnormal signal intensity of the left ventricular subendocardial myocardium indicating myocardial ischemia (Figure 1B, right panel). It is noteworthy that the parents of the patient also underwent physical examinations and echocardiographic assessments conducted by cardiologists, revealing no indications associated with HCM.

### 2.3. Molecular results

We obtained a peripheral blood sample in an EDTA anticoagulant blood sample tube from the patient and stored it at 4°C for less than 6 h. DNA extraction was performed using the Blood Genome Column Medium Extraction Kit (Tiangen Biotech, Beijing, China) according to the instruction. Protein-coding exome enrichment was performed using the xGen Exome Research Panel v1.0. WES was performed using the Illumina NovaSeq 6000 platform (Illumina, San Diego, CA, USA), while primary quality control was performed using FastP, comprising process of the raw data and removement of filter low-quality reads. Variants were annotated in accordance with database-sourced minor allele frequencies (MAFs) and practical guidelines on pathogenicity issued by the American College of Medical Genetics. The sequencing data have been deposited in GSA database (http://ngdc.cncb.ac.cn/gsub/). MutationTaster software and combined annotation dependent depletion (CADD) scaled c-scores were used to predict the pathogenicity of variants, while GRCh37 reference genome was used for alignment. We searched database including gnomAD, ExAC and 1000G to identify prevalence of variants. Effects of genetic variants on protein structure were evaluated via PROVEAN protein batch software with Provean score. As there is no available protein crystal structure for *ALPK3*, AlphaFold database (https://alphafold.ebi.ac.uk/) tool is used to predict protein crystal structure. Within the structure, three important domains have been revealed with analyzed crystal structure. PyMOL software was used to annotate domains and variant sites of the protein. Then we performed modeling analysis and compared three domains with the 6c6m.2.A, 3uto.2.A, and 1ia9.1.A template via SWISS-MODEL database (https://swissmodel.expasy.org/), to visualize and analyze the altered amino acid sequence and stability of *ALPK3*. And other identified variants had been presented in Supplementary Table S1.

**Figure 2:**
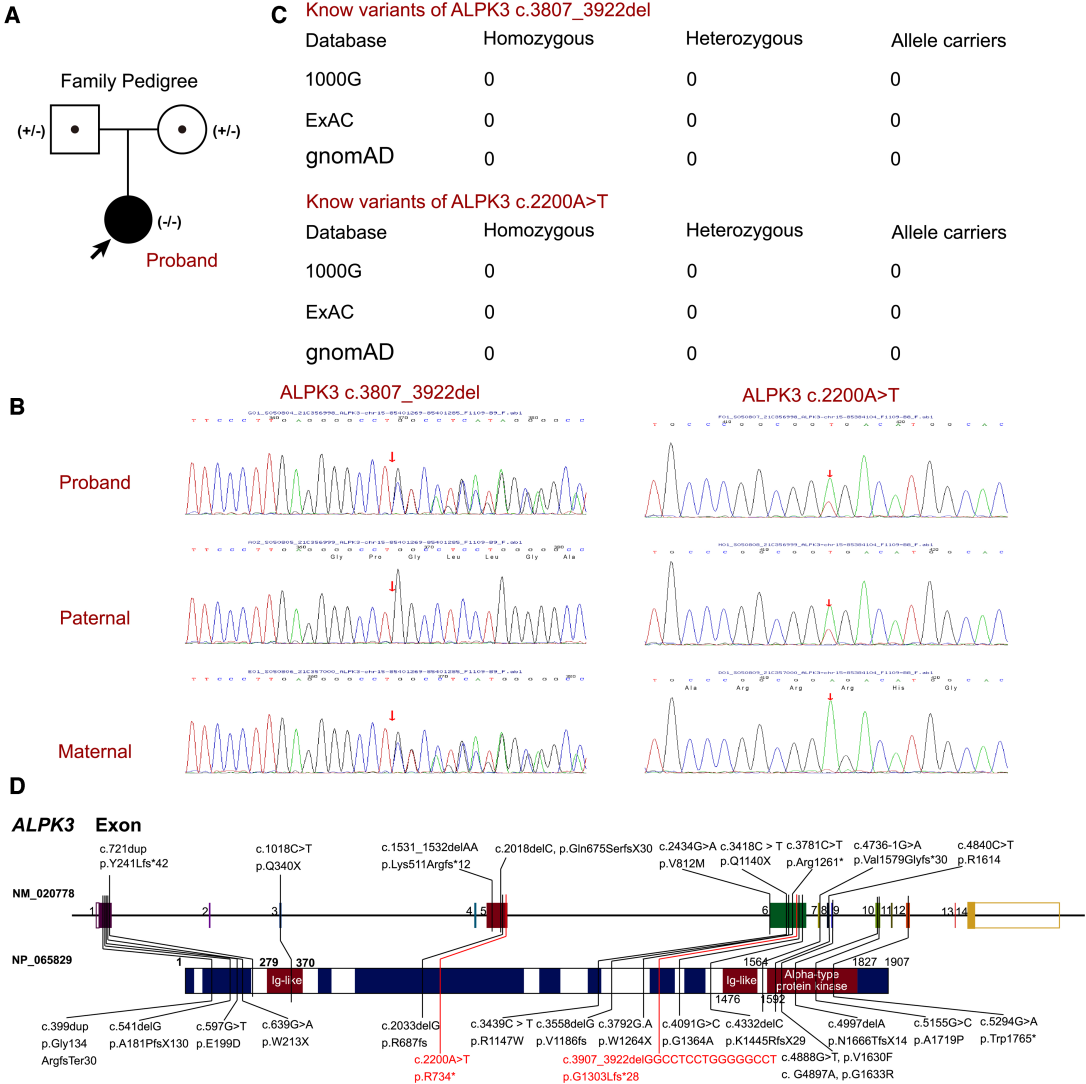
The *ALPK3* variants in this family. (**A**) Family pedigree revealed the maternal carrier of c.3907_3922del (p.G1303Lfs*28) and the paternal carrier of c.2200A>T (p.R734*). The current proband exhibited significant hypertrophic cardiomyopathy with compound heterozygous variants of *ALPK3*. circles represent females, squares represent males, and arrow indicates the proband. Black symbols indicate the clinical presentation of hypertrophic cardiomyopathy, grey symbols indicate carriers. (**B**) Sanger sequencing validation of the current proband and his parents. (**C**) The prevalence of *ALPK3* variants of c.3907_3922del and c.2200A>T. (**D**) Summary of current reports on individual *ALPK3* variants resulting in hypertrophic cardiomyopathy.

Based on the clinical manifestations and laboratory analyses, HCM induced by genetic anomaly was strongly suspected. Thus, WES was performed, which identified a novel compound heterozygous variant of c.3907_3922del (p.G1303Lfs*28) and c.2200A>T (p.R734*) in *ALPK3* gene, while genomic coordinates of these two variants are chr15:85401269-85401285delGGCCTCCTGGGGGCCT and chr15:85384104A>T (depth of coverage is 236.34, percent of exome captured is 98.34%). The patient's parents presented normal cardiac morphology, thus, we employed Sanger sequencing to validate the genotypes of the parents of the patient (forward primer “agcccacacactccttgacc” and reverse primer “tacatcagagctgctgctgg” for c.2200A>T and forward primer “ctgtacctcccgccgcctca” and reverse primer “tcccctgggaacttctcctc” for c.3907_3922del), which revealed that each parent carries a heterozygous variant of the *ALPK3* gene. The variant of c.3907_3922del was maternal inherit, and the variant of c.2200A>T was paternal inherit (Figures 2A,B). According to the American College of Medical Genetics, both variants have pathogenicity as PVS1+PM2_Supporting+PM3 (Trans), and both were related to familial HCM. According to updated data in gnomAD, ExAC and 1000G, these two variants have not been reported in any populations, that means it is the first report of these variants (Figure 2C). Analysis performed with MutationTaster revealed that variant of c.3907_3922del in *ALPK3* was considered pathogenic (probability 1.000) due to nonsense-mediated mRNA decay (NMD), amino acid sequence changed, frameshift, protein features affected and splice site changes, while c.2200A>T was also considered pathogenic because of NMD, acid sequence changed, and protein features affected (probability 1.000). Besides, CADD scaled c-scores of variant c.2200A>T is 36, which implies that the predicted pathogenicity of the variant is extremely high. PROVEAN protein batch software indicated that the p.R734* protein was deleterious with the PROVEAN score of −4.79, due to frameshift and NMD of p.G1303Lfs*28, PROVEAN and SIFT prediction were not applicable. While all the reported variants of *ALPK3* had been listed in Figure 2D.

**Figure 3:**
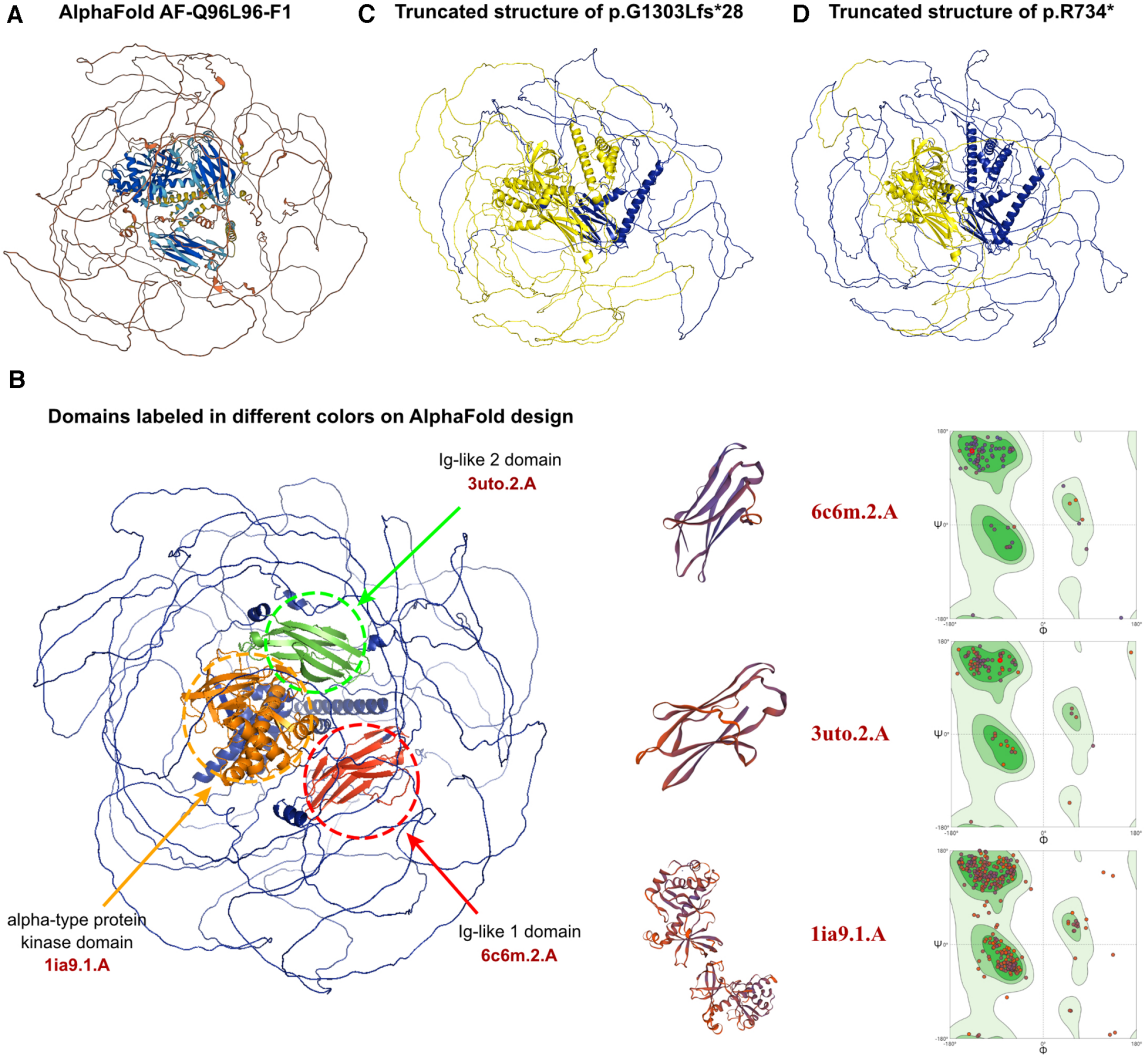
The effects of *ALPK3* c.3907_3922delGGCCTCCTGGGGGCCT and c.2200A>T variants on the molecular structure of the protein. (**A**) AlphaFold protein structure database was used to predict the *ALPK3* wild-type protein crystal structure. (**B**) Three domains of ALPK3 protein were labeled in red, green and orange individually. SWISS-MODEL presented the crystal structures of the variant's three domains, including two immunoglobulin-like (Ig-like) domains and an alpha-type protein kinase domain according to 6c6m.2.A, 3uto.2.A, and 1ia9.1.A model template. Ramachandran plots of three functional domains in wild-type sequences were displayed. (**C,D**) Truncating variant sites of p.G1303Lfs*28 and p.R734* caused by variants of c.3907_3922delGGCCTCCTGGGGGCCT and c.2200A>T was labelled in yellow.

The entire amino acid sequence crystal structure was predicted by AlphaFold and assigned the name AF-Q96L96-F1 (Figure 3A). Although the predicted protein covered the entire length of the amino acid sequence, only three domains demonstrated high confidence (pLDDT >70). These domains were labeled red, green, and orange (Figure 3B). Other regions displayed low confidence in the crystal structure. While all potential templates were searched, only partial parts of the protein had been analyzed previously. We picked structures with the highest predictive value, which may cause several parts do not have a specific folding. In a word, the AlphaFold-predicted structure was the only model that could be utilized. The c.2200A>T and c.3907_3922del variants would result in a protein-truncating variant, typically leading to protein denaturation. The sites of truncated sequences caused by variants were labeled in yellow (Figures 3C,D). SWISS-MODEL was then employed to present the crystal structures of the variant's three domains, including two immunoglobulin-like (Ig-like) domains and an alpha-type protein kinase domain (Figure 3B). An ig-like domain superfamily is a heterogeneous group of proteins that play the role of cell recognition. The alpha-kinase domain is an atypical protein kinase catalytic domain that exhibits no detectable similarity to conventional protein serine/threonine kinases. This protein kinase recognizes protein sequences that adopt an alpha-helical conformation by its initial members, which act as the final link and effector of intracellular information transmission. The identified *ALPK3* variants, c.3907_3922del and c.2200A>T would cause truncated protein, leading to the loss of an Ig-like domain and an alpha-type protein kinase domain, resulting in the dysfunction of the *ALPK3* molecule. The aforementioned analyses suggested that both newly identified variants could alter the transcription of the *ALPK3* gene and damage the protein structures. Therefore, the compound heterozygous variant of *ALPK3* was considered to be genetically associated with HCM in this patient. In addition, several heterozygous variants were identified by WES, such as c.4639A>G in the *FBN1* gene, c.3791G>A in *ANKRD26*, and c.1123G>T in *DPYS*. However, all three genes were considered to exhibit recessive inheritance, and the pathogenicity predictions for these variants were uncertain. Furthermore, all of these variants were inherited from one of her parents, unaffected by associated diseases. Consequently, they were not considered to be associated with HCM.

### 2.4. Treatment and clinical outcome

Following comprehensive laboratory and echocardiographic assessments, the patient was diagnosed with HCM. A 6-week hospitalization period was instituted, during which the patient received a range of medical interventions, including invasive and noninvasive mechanical ventilation, myocardial protection, anti-arrhythmia, cerebral protection, anti-infection, anti-inflammatory, diuresis, and blood transfusion. Although there was residual muscle weakness, the patient was discharged from the hospital after partial recovery from her major concerns with respect to heart rhythm control and cardiac function. However, two weeks after discharge, the patient experienced recurrent cardiac arrest during rehabilitation training and subsequently regained consciousness. The patient was subsequently readmitted to our department, where mechanical ventilation and gastrointestinal decompression were provided to alleviate symptoms. Anti-infective therapy was initiated with cefoperazone and sulbactam, while captopril and metoprolol were prescribed to address HCM and inhibit potentially lethal arrhythmias. Nutritional and rehabilitation therapies were also administered. After a month of comprehensive medical management, including fluid infusion, diuretic therapy, and vitamin supplementation, the patient was discharged with improved symptoms. However, due to the prolonged duration of the condition and recurrent cardiac arrest, the patient had not regained consciousness and experienced severe cognitive and neurological impairment. Oral medication administration and continuous follow-up and evaluation were regularly conducted, with clinic visits scheduled every two weeks in the first month and every three months thereafter.

## 3. Discussion and conclusion

HCM is a primary cardiomyopathy that is commonly associated with a genetic variant. Its prevalence is high worldwide, but some subtypes of HCM with specific genetic variants can result in lethal arrhythmia and severe heart dysfunction, leading to sudden cardiac death (18). It is now commonly accepted that HCM is usually inherited with a complex genetic etiology. Studies and books have revealed that pathogenic variants in the core genes encoding sarcomeric proteins, including thick filament encoding genes *MYBPC3*, *MYH7*, *MYL2*, *MYL3*, and thin filament encoding genes *TNNC1*, *TNNT2*, *TNNI3*, account for over 90% of the pathogenic variants in patients with HCM (19, 20). Additionally, variants in several genes encoding non-sarcomeric proteins with diverse functions, including *ACTN2*, *ALPK3*, *CSRP3*, *FHOD3*, *FLNC*, *JPH2*, *KLHL24*, *PLN*, and *TRIM63*, have also been considered as genetic etiology of HCM (21). Furthermore, variants in *FHL1*, *FXN*, *GAA*, *LAMP2*, and *TTR* genes have an extremely low prevalence of 1/100,000–1/20,000 but have also been reported to be associated with HCM (22).

*ALPK3* gene locates on chromosome 15q25.2 and contains 14 exons. It had been recently identified as a possible disease-causing gene of pediatric HCM, myopathic and dysmorphic skeletal features (10, 17). Initially, the Midori gene was discovered and named by Hosoda et al. through differential display analysis of the P19CL6 cell line (16), and later identified as the *ALPK3* gene. The study revealed that expression of Midori was restricted in the fetal and adult heart and adult skeletal muscle in mice. At the same time, the overexpression of Midori could promote the differentiation of P19CL6 cells into cardiomyocytes (16). A mouse model of *ALPK3* knockout by Van Sligtenhorst et al. in 2012 revealed biventricular hypertrophy in *ALPK3*^−/−^ mice (15). Additionally, the electron microscopy showed impaired cardiomyocyte architecture characterized by reduced numbers of abnormal intercalated discs (15). The experimental data suggested *ALPK3* could regulate the transcript of cardiomyocyte differentiation and heart development, and the loss of function of *ALPK3* would lead to cardiomyopathy. Thus, the OMIM number of HCM in our manuscript is #618052, which is named familial hypertrophic cardiomyopathy-27 caused by homozygous mutation in the *ALPK3* gene (OMIM 617608) on chromosome 15q25. Indeed, several studies have reported on cardiomyopathies caused by other types of kinases. For instance, Brodehl et al. identified protein mutations p.H77Y and p.P70l in integrin-linked kinase, which were found to be associated with arrhythmogenic cardiomyopathy in both humans and transgenic zebrafish (23).

After conducting a comprehensive review of the literature, we identified 22 patients with *ALPK3*-associated HCM, involving 28 distinct variants of the *ALPK3* gene, as described in nine studies and a case report. A summary of all reported variants can be found in Table 1 and Figure 2D (10, 13, 14, 24–29). Consistent with previous reports, the majority of described patients were from consanguineous families, and nearly all patients exhibited biallelic damage, as homozygous or compound heterozygous variants of the *ALPK3* gene were commonly observed (15). Notably, only patients with HCM were identified with heterozygous variants of the *ALPK3* gene, and one such patient was found to have an accompanying DSP gene and was free of lethal cardiac events (29). Thus, *ALPK3* variants appeared to demonstrate a recessive feature in inducing HCM.

**Table 1:** Summarization of reported ALPK3 mutations resulting in hypertrophic cardiomyopathy.

Reference	Mutation site	Genotype	Variant type	Amino acid change	Onset age/gender	Symptom (s)	Extracardiac manifestations	Echo	ECG	Clinical outcome
Almomani et al. 2016	c.4736-1G>A	Hom	Frameshift deletion	p.V1579Gfs*30	At birth/M	Respiratory insufficiency, cyanosis	NA	Severe biventricular dilation	Sinustachycardia, normal PR-interval and QTc, flattened T waves	Dead
	c.3781C>T	Hom	Nonsense mutation	p.R1261*	At birth/F	Generalized hydrops	NA	Severe concentric LV hypertrophy	Biventricular hypertrophy, prolonged QTc, repolarization abnormalities, PVCs	Alive
	c.5294G>A	Hom	Nonsense mutation	p.W1765*	4 y/M	VF, cardiac arrest	Cleft palate, ptosis, low set ears, knee contractures, kyphoscoliosis, talipes equines	Severe concentric LV hypertrophy, RV hypertrophy	VF at age 7, biventricular hypertrophy, prolonged QTc, repolarization abnormalities	ICD implantation
Phelan et al. 2016	c.3792G>A	Hom	Nonsense mutation	p.W1264*	Early infancy/F	Echo/ECG anomaly	Cleft palate, intraoral pterygia, knee and shoulder contractures, camptodactyly, webbed neck	Severe hypertrophy of the LV and IVS	Prolonged QT, SVT, nsVT	ICD implantation
Çağlayan et al. 2017	c.2018delC	Hom	Frameshift deletion	p.Q675Sfs*30	21 w of gestation/M	Echo anomaly	Low set ears, high arched palate	Diffuse LV hypertrophy	NA	Alive
Jaouadi et al. 2018	c.1531_1532delAA	Hom	Frameshift deletion	p.K511Rfs*12	7 d/M	Respiratory distress	Cleft palate, ptosis, low set ears, micrognathia, camptodactyly, webbed neck, knee stiffness	Concentric LV hypertrophy	NA	Unknown
Al Senaidi et al. 2019	c.639G>A	Hom	Nonsense mutation	p.W213*	At birth/M	Tachypnea, heart failure	Low set ears, high arched palate	Biventricular hypertrophy	LV hypertrophy	Alive
Herkert et al. 2020	c.1018C>T	Comp het	Nonsense mutation	p.Q340*	No.1: At birth/M No.2: At birth/M No.3: 4 m/F	Echo/ECG anomaly	No.1: Ptosis, ankyloglossia, hypertelorism, low set ears, micrognathia, knee contractures, webbed neck No.2: Hypertelorism, low set ears, micrognathia, knee contractures, webbed neck No.3: NA	No.1: Significant LV hypertrophy, mild RV hypertrophy No.2: Biventricular hypertrophy No.3: Biventricular hypertrophy	No.1: Prolonged QTc No.2: Prolonged QTc No.3: Biventricular hypertrophy, prolonged QTc, VF at age 11	All alive
	c.2434G>A	Comp het	Missense mutation	p.V812M	No.1: At birth/M No.2: At birth/M					
	c.4332delC	Comp het	Frameshift deletion	p.K1445Rfs*29	No.3: 4 m/F					
	c.541delG	Comp het	Frameshift deletion	p.A181Pfs*130	31 y/M	Sinus bradycardia	Hypertelorism	Biventricular dilation	Sinusbradycardia, high-voltage QRS complex, normal QTc, 2nd degree AV block	Alive
	c.3439C>T	Comp het	Missense mutation	p.R1147W						
	c.4997delA	Comp het	Frameshift deletion	p.N1666Tfs*14	53 y/M	Echo/ECG anomaly	Spondylolysis, unilateral hearing loss (conductive)	Asymmetric LV hypertrophy	SR, LV hypertrophy, short PR without pre-excitation but increased PR dispersion, prolonged QTc at high heart frequencies, repolarization abnormalities, nsVT (once)	Alive
	c.4091G>C	Comp het	Missense mutation	p.G1364A						
	c.5105+5G>C	Comp het	Missense mutation	p.(?)	No.1: 3 m/F No.2: 3 m/F	Echo/ECG anomaly	No.1: bilateral hearing loss (conductive) No.2: NA	Moderate progressive LV hypertrophy	SR, LV hypertrophy, short PQ interval, prolonged QTc, repolarization abnormalities	All alive
	c.597G>T	Comp het	Missense mutation	p.E199D						
	c.4888G>T	Comp het	Missense mutation	p.V1630F	14 y/F	Echo/ECG anomaly	Cleft palate, high arched palate, low set ears, camptodactyly, kyphoscoliosis, webbed neck	Severe concentric LV hypertrophy, moderate RV hypertrophy	Extreme septal hypertrophy, prolonged QTc, repolarization abnormalities	ICD implantation
	c.2023delC	Comp het	Frameshift deletion	p.Q675Sfs*30						
	c.3418C>T	Hom	Nonsense mutation	p.Q1140X	9 y/F	Echo/ECG anomaly	Cleft palate, camptodactyly, kyphoscoliosis	Concentric LV hypertrophy	SR, biventricular hypertrophy, prolonged QTc, repolarization abnormalities	Alive
	c.5155G>C	Hom	Missense mutation	p.A1719P	35 w/F	Echo/ECG anomaly	Cleft palate, hypertelorism, low set ears, micrognathia, webbed neck	Concentric LV hypertrophy, moderate RV hypertrophy	Biventricular hypertrophy, repolarization abnormalities	Alive
Jorholt et al. 2020	c.2033delG	Comp het	Frameshift deletion	p.R687fs	6 m/M	Respiratory distress, heart failure	Broad forehead, cleft palate, axial hypotonia, webbed neck, pectus excavatum, scoliosis	Asymmetric LV hypertrophy	prolonged QTc, repolarization abnormalities	Dead
	c.3558delG	Comp het	Frameshift deletion	p.V1186fs						
	c.4897G>A	Hom	Missense mutation	p.G1633R	21 y/F	Palpitations, dyspnea, heart failure	NA	Severe LV and IVS hypertrophy	Severe LV hypertrophy, prolonged QTc	Heart transplantation
Ding et al. 2021	c.721dup	Comp het	Frameshift deletion	p.Y241Lfs*42	10 m/F	Echo anomaly	Cleft palate, low set ears, scoliosis, knee contractures, webbed neck	LV and IVS hypertrophy	LV hypertrophy, ST-T changes in multiple-lead, T wave inversion, prolonged QTc	Unknown
	c.4840C>T	Comp het	Nonsense mutation	p.R1614*						
Carlo et al. 2022	c.399dup	Het	Frameshift deletion	p.G134Rfs*30	60 y/M	Chest pain, palpitations	NA	Concentric LV hypertrophy	Left anterior hemiblock, premature atrial contractions	Alive
This study	c.3907_3922delGGCCTCCTGGGGGCCT	Comp het	Frameshift deletion	p.G1303Lfs*28	14 y/F	Respiratory distress, fatigue	Low set ears, scoliosis	Diffuse biventricular hypertrophy	Biventricular hypertrophy, ventricular escape	Alive
	c.2200A>T	Comp het	Nonsense mutation	p.R734*						

Comp het, compound heterozygous; Echo, echocardiography; ECG, Electrocardiograph; F, female; Het, heterozygous; Hom, homozygous; ICD, implantable cardioverter defibrillators; IVS, interventricular septum; LV, left ventricle; m, month(s); M, male; nsVT, non-sustained ventricular tachycardia; NA, not available; RV, right ventricle; SR, sinus rhythm; SVT, supraventricular tachycardia; VF, ventricular fibrillation; w, week(s); y, year(s).

The clinical presentation of *ALPK3*-associated HCM varied, with most pediatric patients presenting symptoms or positive imaging results before age 18. In addition to typical clinical manifestations and findings on ECG and echocardiography, extracardiac manifestations, such as facial and musculoskeletal abnormalities, were observed in some patients (10, 24, 25, 27). Fatal arrhythmia, such as ventricular fibrillation, was the leading cause of death, necessitating ICDs and, in some cases, heart transplantation. Notably, only nonsense and frameshift variants among the 22 reported patients resulted in death and fatal arrhythmia, necessitating ICD implantation. Conversely, missense variants were associated with mild clinical outcomes. Thus, homozygous and compound heterozygous variants of *ALPK3* with nonsense or frameshift variants were linked to adverse clinical outcomes, warranting careful follow-up and timely ICD implantation to prevent sudden cardiac death. Therefore, given the patient's clinical symptoms and history of cardiac arrest, we recommended ICD implantation. Regrettably, the patient's family declined this recommendation.

In the present study, we report the case of a 14-year-old female who suffered from *ALPK3*-associated HCM and experienced sudden cardiac arrest. Through molecular analysis, we identified a novel compound heterozygous variant (c.3907_3922del and c.2200A>T) in the *ALPK3* gene, which was inherited from her parents and resulted in truncated protein formation. WES was employed for molecular diagnosis, which has emerged as an efficient and favorable technique for HCM diagnosis and provides valuable insights for assessing sudden cardiac death risks. The entire process, from sampling to library preparation for sequencing, usually takes about five days, and subsequent analysis requires approximately one week, enabling us to produce a report within two weeks. Therefore, we recommend the utilization of WES for the timely identification of deleterious variants in HCM patients. Furthermore, our findings suggest that the nonsense and frameshift variants of *ALPK3* are associated with unfavorable clinical outcomes, and prompt implantation of an ICD is crucial for preventing sudden cardiac death.

## Data Availability

The datasets presented in this study can be found in online repositories. The names of the repository/repositories and accession number(s) can be found in the article/**Supplementary Material**.
