# Supplementary material for: A novel compound heterozygous variant in ALPK3 induced hypertrophic cardiomyopathy: a case report
Source: Front Cardiovasc Med. 2023 Jun 15;10:1212417. doi: 10.3389/fcvm.2023.1212417 (PMC10311070; doi:10.3389/fcvm.2023.1212417)
Supplement: Supplementary file 1 [file Table1.docx]

**TABLE S1. Other genetic variants present in the investigated patients**

| **Gene** | **Chromosomal location** | **Transcript**  **(Exon)** | **Nucleotide**  **(Amino Acid)** | **Genotype** | **Normal Population Frequency** | **Pathogeniciy Prediction** | **Pathogeniciy Analysis** | **Phenotype**  **(Mode of Inheritance)** |
| --- | --- | --- | --- | --- | --- | --- | --- | --- |
| *DPYS* | chr8:105436587 | NM_001385  (exon7) | c.1123G>T  (p.V375L) | Het | 0.0001 | LD | Uncertain | Dihydropyrimidine Dehydrogenase Deficiency (AR) |
| *SLC4A1* | chr17:42336868 | NM_000342  (exon8) | c.691G>C  (p.V231L) | Het | 0.0000544 | D | Uncertain | 1. Autosomal Dominant Distal Renal Tubular Acidosis (AD)  2. Autosomal Recessive Distal Renal Tubular Acidosis (AR)  3. Spherocytic Anemia SA type (AD)  4. Stomatocytosis (AD)  5. Hereditary Spherocytosis type 4 (AD) |
| *SLC45A2* | chr5:33954545 | NM_016180  (exon4) | c.953G>A  (p.R318H) | Het | 0.0003994 | U | Uncertain | 1. Skin Hair Eye Pigmentation Variation Type 5 (AR)  2. Oculocutaneous Albinism Type 4 (AR) |
| *F10* | chr13:113783907 | NM_000504  (exon2) | c.212T>C  (p.F71S) | Het | 0.0001 | D | Pathogenic | Factor Ⅹ Deficiency (AR) |
| *F8* | chrX:154088883 | NM_000132  (exon25) | c.6724G>A  (p.V2242M) | Het | 0.0067826 | U | Uncertain | 1. Hemophilia A (XLR)  2. Factor Ⅷ Deficiency (XLD) |
| *MUC1* | chr1:155160317 | NM_001204285  （exon6） | c.962T>C  (p.V321A) | Het | 0.0001 | U | Uncertain | Autosomal Dominant Tubulointerstitial Kidney Disease Type 2 (AD) |
| *AP3B1* | chr5:77477362 | NM_003664  (exon8) | c.911C>T  (p.T304I) | Het | 0.0008166 | U | Uncertain | Hermansky-Pudlak Syndrome Type 2 (AR) |
| *RPL3L* | chr16:2002996 | NM_005061  (exon3) | c.244C>G  (p.P82A) | Het | 0.000173 | U | Uncertain | Dilated Cardiomyopathy Type 2D (AR) |
| *MYO6* | chr6:76623908 | NM_004999  (exon34) | c.3568A>C  (p.K1190Q) | Het | 0.0001 | U | Uncertain | 1. Autosomal Recessive Deafness 37 (AR)  2. Autosomal Dominant Deafness 22 (AD) |
| *CYP4V2* | chr4:187117196 | NM_207352  (exon3) | c.367A>G  (p.M123V) | Het | 0.00087528 | U | Uncertain | Bietti's Crystalline Corneoretinal Dystrophy (AR) |
| *TTC37* | chr5:94877061 | NM_014639  (exon7) | c.350A>T  (p.D117V) | Het | 0.0001 | U | Uncertain | Trichohepatoenteric Syndrome 1 (AR) |
| *GRHL3* | chr1:24658038 | NM_198174  (exon2) | c.140T>C  (p.M47T) | Het | 0.0002175 | U | Uncertain | Van-Der-Woude Syndrome Type 2 (AD) |
| *ESPN* | chr1:6485232 | NM_031475  (exon1) | c.217C>G  (p.P73A) | Het | 0.0000896 | U | Uncertain | 1. Autosomal Recessive Deafness type 36 (AR)  2. Autosomal Dominant Deafness(AD) |
| *CC2D2A* | chr4:15591226 | NM_001080522  (exon34) | c.4238G>A  (p.C1413Y) | Het | 0.00051348 | U | Uncertain | 1. Meckel Syndrome type 6 (AR)  2. Joubert Syndrome type 9 (AR)  3. COACH Syndrome type 2 (AR)  4. Retinitis Pigmentosa 93 (AR) |
| *TCF20* | chr22:42575689 | NM_005650  (exon3) | c.5675A>C  (p.E1892A) | Het | 0.00019231 | U | Uncertain | Developmental Delay with Intellectual Disability and Behavioral Abnormalities (AD) |

Het= heterozygous; LD= likely dangerous; D= dangerous; U= uncertain; AD= autosomal dominant inheritance; AR= autosomal recessive inheritance; XLD= X-linked dominant inheritance; XLR= X-linked recessive inheritance.
